# Shock-induced breaking of the nanowire with the dependence of crystallographic orientation and strain rate

**DOI:** 10.1186/1556-276X-6-291

**Published:** 2011-04-05

**Authors:** Fenying Wang, Yajun Gao, Tiemin Zhu, Jianwei Zhao

**Affiliations:** 1 Key Laboratory of Analytical Chemistry for Life Sciences, Ministry of Education, School of Chemistry and Chemical Engineering, Nanjing University, Nanjing 210008, P. R. China

## Abstract

The failure of the metallic nanowire has raised concerns due to its applied reliability in nanoelectromechanical system. In this article, the breaking failure is studied for the [100], [110], and [111] single-crystal copper nanowires at different strain rates. The statistical breaking position distributions of the nanowires have been investigated to give the effects of strain rate and crystallographic orientation on micro-atomic fluctuation in the symmetric stretching of the nanowires. When the strain rate is less than 0.26% ps^-1^, macro-breaking position distributions exhibit the anisotropy of micro-atomic fluctuation. However, when the strain rate is larger than 3.54% ps^-1^, the anisotropy is not obvious because of strong symmetric shocks.

## Introduction

In recent years, the metallic nanowires applied as nanoconnectors [1] and the active components of nanoelectromechanical system (NEMS) devices [2,3] have attracted extensive interests owing to their special mechanical [4], thermal [5], electrical [6], and magnetic [7] properties. The approaches to investigate nanowires in experiments include using scanning tunneling microscopy (STM) [8,9], atomic force microscopy (AFM) [10], transmission electron microscope (TEM) [11,12], and mechanically controllable break junctions (MCBJ) [13,14]. However, it is difficult to manipulate the deformation processes when the nanowires are applied in NEMS, because controlling the failure of the nanowires is a challenging thing due to their small scales. Hemker [15] proposed the reliability of NEMS would require a fundamental description of its deformation mechanism, which must be based on a corresponding solid understanding. In contrast, molecular dynamics (MD) simulation [16,17], which solves Newton's equations of motion for a collection of interacting particle over a number of time steps, is an effective method to study the deformation and breaking failure processes of the metallic nanowires.

With the method of MD simulation, Koh and Lee [18] studied the strain-rate effects on the tensile structure of platinum nanowire. Meanwhile, they [19] gave the mechanical behaviors of gold and platinum nanowires under different strain rates, which indicated that the displayed crystalline-ordered deformation of the nanowires was governed by the formation of a main dislocation plane at low strain rate. Ikeda et al. [20] proposed amorphization in nickel nanowire induced by high strain rate. These studies indicate strain-rate effects on the deformation of the single-crystal metallic nanowires. For the single-crystal materials, we noticed that the plastic response in copper could occur rapidly [21,22]. According to this point, studying the deformation and breaking failure of the copper single-crystal nanowires shall be of vital importance for developing and processing the nanoscale systems based on metallic nanowires. In addition, anisotropies in single-crystal materials will give rise to the dependence of crystallographic orientation. For example, Tsuru and Shibutani [23] showed copper had a much larger anisotropic factor than aluminum in terms of the load-depth relation and stress distribution. Bringa et al. [24] proved single-crystal copper had a marked anisotropic behavior in shock wave propagation. It is known that crystallographic orientation is related with structural anisotropy and symmetric stretching at different strain rates will generate different mechanical shocks, but we do not know which factor will dominate the deformation and the breaking failure mechanism of the nanowires? In order to make the question clear, we focused on the MD simulation investigation of the tensile deformation and breaking failure of the single-crystal copper nanowires under the effects of crystallographic orientation and strain rate.

**Figure 1:**
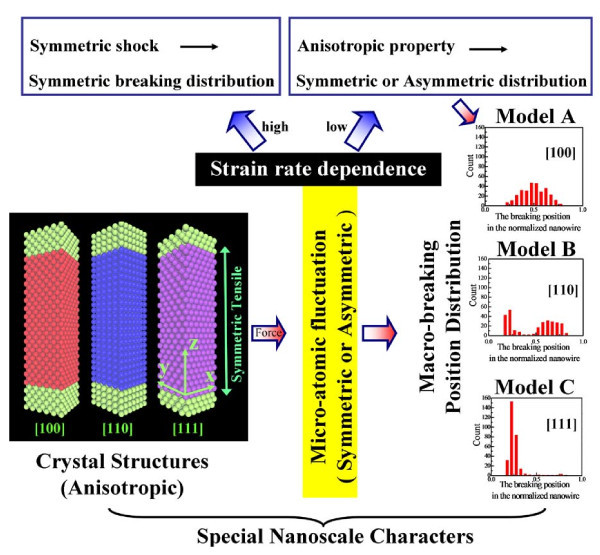
**Schematic illustration of the theoretical models**.

It is also worth noting that the nanowires not only behave as intrinsic properties like bulk materials, but also have their special microcosmic behaviors due to nanoscale effects. In our previous work [25,26], the breaking uncertainty of the nanowire was found when the nanowire was stretched at different lengths and strain rates. The similar microscopic phenomena in experiments were also found in the metallic and the molecular junction conductance followed by a statistical distribution [27,28]. In our study, we designed different initial equilibrium states to investigate statistically the [100], [110], and [111] single-crystal copper nanowires. As shown in Figure 1, we propose a theoretical explanation for the relationship of micro-atomic fluctuation and macro-breaking position distribution based on symmetric stretching and structural anisotropy. We find micro-atomic fluctuation plays a critical role in the deformation of nanowires. At low strain rates, macro-breaking position distributions reflect anisotropic characters of the single-crystal nanowires, whereas the anisotropic characters behave unobvious at high strain rates because shocks induced by strong symmetric stretching dominate the breaking failure at two ends of the nanowires.

## Methodology

To reveal the relationship between anisotropy and symmetric mechanical shocks, MD simulations were performed to study a total samples of 14,400 (3 × 16 × 300) of the single-crystal copper nanowires. The geometric models of face-centered cubic (FCC) were generated as a regular lattice along the [100], [110], and [111] crystallographic orientations, respectively. The geometric dimension of nanowires in the simulations was set as 5*a *× 5*a *× 15*a *(*a *stands for lattice constant, 0.362 nm for copper), corresponding to 1,500 atoms. For each orientation, total of 16 strain rates were set from 0.01 to 7.69% ps^-1^, corresponding to the absolute rates from 0.08 to 481.07 m/s in Table 1. Different and enough time was adopted to relax the single-crystal copper nanowires to get 300 initial equilibrium states for one crystallographic orientation at each strain rate.

**Table 1:** The applied strain rate and its absolute rate of the nanowires

**No**.	Applied strain rate (% ps^-1^)	The absolute rate (m/s)	**No**.	Applied strain rate (% ps^-1^)	The absolute rate (m/s)
<1>	0.01	0.08	<9>	2.31	125.43
<2>	0.03	1.67	<10>	3.08	167.23
<3>	0.08	4.18	<11>	3.54	192.22
<4>	0.15	8.36	<12>	4.08	221.54
<5>	0.26	13.94	<13>	4.62	250.84
<6>	0.51	27.81	<14>	5.39	292.68
<7>	0.76	41.81	<15>	6.16	334.45
<8>	1.54	83.61	<16>	7.69	418.07

The single-crystal copper nanowires were subjected to uniaxial strain by uniformly moving the top and bottom fixed layers in the *z*-direction (Figure 1). The strain (*ε*) was defined as *ε *= (*l*-*l*_0_)/*l*_0_, where *l *was the current stretching length and *l*_0 _was the length just after relaxation. Free boundary condition was adopted. The Verlet leapfrog algorithm was used for the integration of motion equations to obtain velocity and trajectories of atoms. Nośe-Hoover thermostat [29-31] as a rescaling method of velocity maintains the system at 300 K. The interaction between copper atoms was described through embedded-atom method (EAM) potential function developed by Johnson [32-34], which could provide an effective description of the transition metals with the FCC structure. The total energy was given by:(1)(2)

where *E *is the total internal energy of the system, *V *is the pair potential between atoms *i *and *j*, and *r_ij_*is the distance between them, *F*(*ρ_i_*) is the energy to embed atom *i *in an electron density *ρ_i_*, *φ*(*r_ij_*) is the electron density at atom *i *due to atom *j *as a function of the distance *r_ij_*. The stress (*σ*) in *z*-direction was calculated by the Virial scheme [35] as following:(3)

Where  is the stress tensor of atoms *α *in the tensile direction (*z*-axis), *Ω_i_*is the volume of *i *atoms, *m *is the mass, and  is the velocity component of atom *i *in the *z-*direction. *ϕ*, *F*, *ρ*, and *ƒ *are parameters from EAM potential [32], which corresponding to the pair potential, the embedded energy, the electron density between the atom *i *or *j *and all other atoms, the electron density in *r_ij_*between atomic *i *and *j*, respectively. The first and second terms in the right side of the above equation represent the thermal effect and the atomic interactions, respectively. All the presented MD simulations and visualization process were performed with the self-developed software NanoMD [36], the reliability of algorithms has been validated not only by a large amount of theoretical simulations [25,26,37-41], but also with the comparison to the experimental measurements [42,43].

## Results and discussions

It is shown in Figure 1 that macro-breaking position distributions of the nanowires are from the micro-atomic fluctuation of three crystallographic orientations at different strain rates and the ways of atomic fluctuation are related with deformation mechanism of the nanowires. With the MD simulations of the nanowires at the strain rates from 0.01 to 7.69% ps^-1^, Videos S1-S3 in Additional files 1, 2 and 3 are selected to exhibit the representative deformation behaviors of the [100] single-crystal copper nanowires at the strain rates of 0.01, 1.54, and 6.16% ps^-1^, respectively. At low strain rate of 0.01% ps^-1^ (Video S1 in Additional file 1), the nanowire slips along (111) planes after the elastic deformation. In general, for the FCC closed pack structure, Burgers vectors exit along the <110> direction and induce the structure slip and reconstruct themselves along (111) planes. The slippage mechanism had been discussed in detail by Finbow et al. [44], who gave that the overall dislocation associated with slippage had a Burgers vectors given by (*a*_0_/2) [] in the nanoscale wire. The process can be better described as a uniform slip in the [] direction of one (111) plane relative to the neighboring one. Slippage retains the crystalline order in the plastic deformation, and the linear atomic chains tend to occur near the middle of the nanowire because of symmetric stress. At 1.54% ps^-1^ (Video S2 in Additional file 2), the obvious slippage allowing for reconstruction along (111) plane is not found and the nanowire shows superplasticic behavior with amorphous structures. At 6.16% ps^-1^ (Video S3 in Additional file 3), the nanowire is more likely to break near the two ends because of local melted structures.

**Figure 2:**
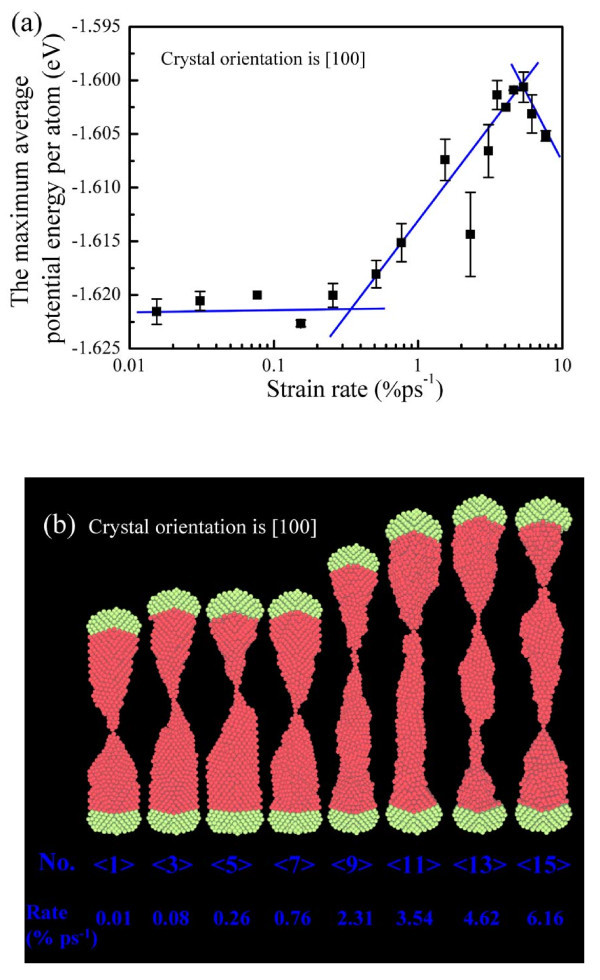
**The potential energy and corresponding deformation of the [100] single-crystal copper nanowire**. **(a)** The maximum average potential energy per atom of the [100] nanowire plotted against strain rates, **(b)** the representative snapshots of the [100] copper nanowire.

The deformation styles of the [100] single-crystal copper nanowires are mainly slippage, amorphization, and local melted structures at low, middle, and high strain rates, respectively. The dependence of deformation characters at the applied strain rates is related with the micro-atomic fluctuation of crystalline structures. The degree of lattice order could be reflected by the maximum average potential energy per atom, which results from the breaking of metallic bonds in the tensile deformation process. Bond breaking is a direct consequence of atomic fluctuation overcoming the interatomic cohesive energy, which in turn causes the disordered fluctuation of atoms. When the nanowire stretches, the atoms under strong shocks overcome the interatomic cohesive energy to get a disordered amorphous state with the increase of the average potential energy per atom. Figure 2a shows the maximum average potential energy per atom at each strain rate from the statistical results, and the energy increases with the strain rate increasing before the high strain rate of 5.39% ps^-1^, but it exhibits a decreasing trend after the strain rate of 5.39% ps^-1^, attributing that symmetric shocks are so strong that the shock wave at such high strain rate (>5.39% ps^-1^) has some difficulties in propagating from the two ends to the middle of the nanowire. So the nanowire behaves as the local melted structures at the two ends and retains the order lattice within the nanowire. We can also see the corresponding breaking characters at the selected strain rates from the representative snapshots of the [100] single-crystal copper nanowires at the breaking moment in Figure 2b.

In comparison with [100], the [110] single-crystal copper nanowires behave as different deformation behaviors at the strain rates of 0.01, 1.54, and 6.16% ps^-1^ (see Videos S4-S6 in Additional files 4, 5 and 6). As shown in Video S4 in Additional file 4, the [110] nanowire prefers to maintain the crystallographic structure at low strain rate of 0.01% ps^-1^. The neck appears abruptly with the strain increasing, and the nanowires break accompanying with a few atoms in the disorder movement. This behavior is in agreement with the observations of Tavazza et al. [45] Because the preferred slip directions are identical to the tensile direction so the system has no ability to get the atomic rearrangement at lower strain rates. The nanowires remain a better crystal structures during the weak mechanical shocks at low strain rates. Increasing the strain rate will result in the increasing of atomic thermal motion, which facilitates the ductility of the materials. The deformation behavior in Video S5 (Additional file 5) shows that a local lattice reconstruction becomes predominant after the first yield point and the necking takes place at the positions of the lattice reconstruction. Unlike at the low and middle strain rates, the [110] nanowire at the strain rate of 6.16% ps^-1 ^(Video S6 in Additional file 6) exhibits superplasticity behavior with local disordered deformation. With the tension strain increasing, the [110] nanowire is more likely to break near the two ends of the nanowire due to the symmetric stress and local melted structures.

For the special deformation behaviors of the [111] single-crystal copper nanowires at the strain rates of 0.01, 1.54, and 6.16% ps^-1^ (see Videos S7-S9 in Additional files 7, 8 and 9), the difference from [100] and [110] is that the deformation mechanism is the partial lattice rotation for the [111] nanowires. After the relaxing, the nanowire retains relative order lattice at the low strain rate of 0.01% ps^-1^, and the disorder crystal structures becomes obvious at the strain rate of 1.54% ps^-1^. When the strain rate is at 6.16% ps^-1^, the local disorder structures distribute at the two ends of the nanowires with the strain increasing, not at one side of 0.01% or 1.54% ps^-1^. In the stretching processes of the nanowires, the disorder crystal structures increase obviously with the strain rate increasing. The effects of strain rates on deformation structures could be reflected by the maximum average potential energy per atom in Figure S1 (Additional file 10), which increases generally for each crystallographic orientation with the strain rate increasing. Within the simulated strain rates, the [111], [100], and [110] nanowires have the lowest energy, the intermediate energy, and the highest energy, respectively, which are consistent with lattice plane energies in FCC metals [46,47].

From the above results, the single-crystal copper nanowires present various deformation behaviors at each crystallographic orientation. At low strain rates, clear slippage for [100] orientation occurs along the (111) planes. When the copper nanowires are stretched along the [110] orientation, the obvious lattice reconstruction becomes predominant after the first yielding point, whereas the [111] nanowires are partial lattice rotation in the deformation processes. However, at high strain rates, the nanowires always behave as local disorder structures at the two ends for each crystalline orientation. The dependence of deformation mechanism on strain rate and crystallographic orientation indicates that anisotropy behaves obvious at low strain rates, whereas unobvious at high strain rates due to strong symmetric stretching. Different deformation behaviors of the nanowires, which may be effectively evaluated by the mechanical property, are attributed to the different deformation mechanisms.

**Figure 3:**
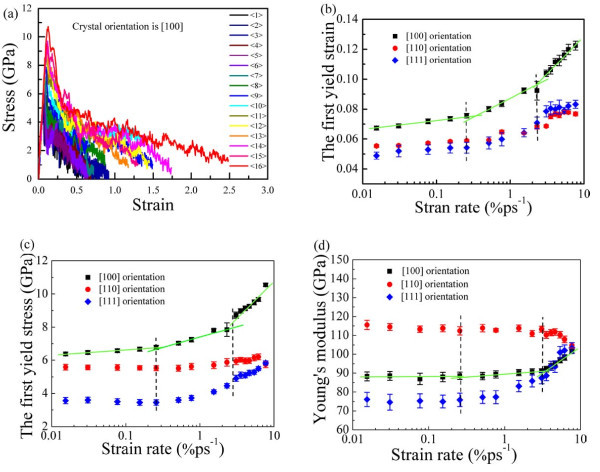
**The mechanical behavior of the single-crystal copper nanowire**. **(a)** The representative stress-strain relationship of the [100] copper nanowire at the strain rates from 0.01 to 7.69% ps^-1^. **(b)** The first yield strain of [100], [110], and [111] plotted against strain rates, respectively. **(c)** The first yield stress of [100], [110], and [111] plotted against strain rates. **(d)** Young's modulus of [100], [110], and [111] plotted against strain rates.

For the effect of the strain rate on the mechanical property, Figure 3a shows the typical stress-strain responses of the [100] single-crystal copper nanowires at strain rates from 0.01 to 7.69% ps^-1^ (to refer to strain rates in Table 1). Stress increases linearly with the strain increasing before the first yield point (the critical point between elastic and plastic deformation), which is consistent with elastic law (That is *σ*_1_ = *Yε*_1_, *σ*_1_, and *ε*_1 _are the first yield strain and stress, respectively. *Y *is Young's modulus.). When the stress decreases abruptly after the first yield point, the nanowire undergoes an irreversible deformation which indicates the beginning of plastic deformation. Subsequently, the yield cycle repeats continuously with a decreasing trend, and the yield cycle is over when the nanowires have no ability to maintain their structures and finally break. At low strain rates, the displayed periodic characters of the stress-strain responses imply the presence of the temporary stable state. By contrast, the periodicity at high strain rates is not obvious in the whole yield cycle.

For the dependence of crystallographic orientation, Figure 3b, c and 3d give the first yield stress, strain, and Young's modulus as a function of strain rate, respectively. Strain rates applied on the [100], [110], and [111] nanowires are all from 0.01 to 7.69% ps^-1^, and the average statistical result is from 300 samples for each strain rate. For the [100] single-crystal copper nanowires, the first yield strain and stress both increase with the strain rate increasing. However, the first yield strain and stress are insensitive to the lower strain rate, whereas they are sensitive to the higher strain rate. Here, we named the divided range of strain rates as insensitive area (I), transitional area (II), and sensitive area (III). Young's modulus (*Y*) is defined by Sun [48] as the stress of a material divided by its strain in the elastic deformation region, which may be used to evaluate the mechanical strength of the nanowires. In the insensitive area of strain rates (I), the average *Y *fluctuates within its error, and it behaves as an increasing trend in the transition area of strain rates (II). While reaching the sensitive area of strain rates (III), *Y *abruptly increases in a line with the strain rate increasing, indicating the presence of the hardening effect. However, while comparing among [100], [110], and [111] crystallographic orientations, the mechanical properties of the [110] nanowires behave as different characters. It is attributed to the deformation mechanism and breaking behavior of the [110] nanowire at different strain rates, i.e., the [110] nanowire always prefers to maintain its crystallographic structure at low, middle, and high strain rates, respectively, (see Videos S4-S6 in Additional files 4, 5 and 6), so the mechanical property behaves insensitively to strain rates. In general, the stress and the *Y *of the [110] are not sensitive to strain rates within the range of strain rates, but the *Y *of the [110] indicates the largest mechanical strength.

The structural anisotropy and symmetric stretching under different strain rates could bring different deformation mechanisms and mechanical properties, which could give insight into mechanical breaking failure and operation of metallic nanowires. If we could predict the deformation behaviors and the final breaking positions of the nanowires, the breaking failure could be controlled and the nanowires also could be strengthened near the breaking positions to avoid failure.

**Figure 4:**
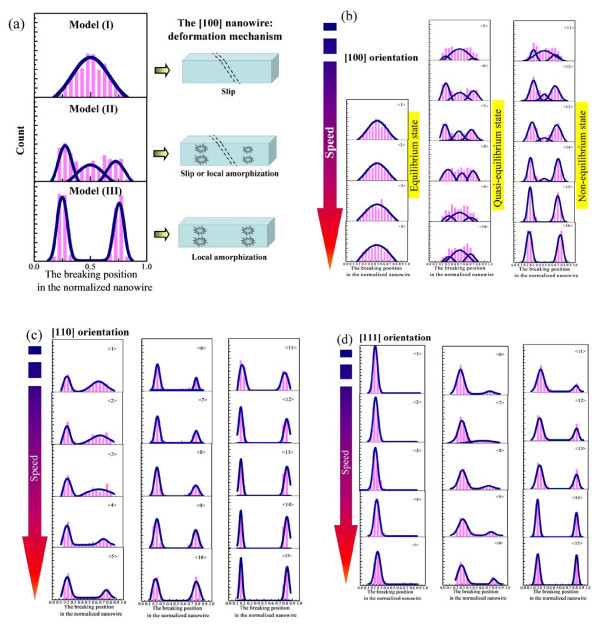
**The breaking position distribution of the single-crystal copper nanowire**. **(a)** Models of the breaking position distribution (I), (II), and (III) of the [100] nanowire at three representative strain rates, and schematic illustration of the corresponding deformation mechanism of the [100] copper nanowire, **(b)** the breaking position distributions of the [100] nanowires at all the simulated strain rates from 0.01 to 7.69% ps^-1^, **(c)** The breaking position distributions of the [110] nanowires at all the simulated strain rates from 0.01 to 6.16% ps^-1^, **(d)** The breaking position distributions of the [111] nanowires at all the simulated strain rates from 0.01 to 6.16% ps^-1^.

For example, in most cases, the final breaking positions of [100] occur at the central part of the nanowire at low strain rates, and the nanowires are apt to break at the two ends with the strain rate increasing. Using the [100] nanowire, the scheme in Figure 4a shows the relationships between macro-breaking position distribution and deformation mechanism induced by micro-atomic fluctuation. The statistical histograms of the breaking positions are fitted with Gaussian function, and the fitting peaks replace the most probable breaking position (MPBP) of the nanowires [25]. The MPBP is in the middle of the [100] nanowire at the insensitive area of strain rate (I), and the MPBP distributes at the two ends of the [100] nanowire at the sensitive area of strain rate (III), whereas the MPBP is in the middle or two ends of the [100] nanowire at the transition area of the strain rate (II). In detail, Figure 4b shows the MPBP distributions of the [100] nanowires at the strain rates from 0.01 to 7.69% ps^-1^ (to refer to strain rates in Table 1). The breaking position distributions at three areas correspond to the stretching deformation processes of the nanowires in equilibrium state, quasi-equilibrium state, and non-equilibrium state, respectively. At low strain rates, the slippage along (111) planes dominates the stretching deformation, and atomic fluctuation is in an equilibrium state. Strong shocks at high strain rates result in the superplastic behaviors and local melted structures, which induced atomic fluctuation in non-equilibrium state, whereas the atomic fluctuation in quasi-equilibrium state brings irregular character of the MPBP distribution at the transition area of the strain rate (II). It reflects the microscopic uncertain property of nanoscale materials. Moreover, there is a transition among the equilibrium state, quasi-equilibrium state, and non-equilibrium state, e.g., Figure 〈11〉 in Figure 4b belongs to quasi-equilibrium state of non-equilibrium state.

Under the same simulation conditions of the [100] nanowires, the breaking position distributions of the [110] and [111] nanowires behave completely different at the strain rates from 0.01 to 7.69% ps^-1 ^(to refer to strain rates in Table 1). The breaking position distributions in Figure 4c show that the [110] nanowires would like to break at two ends of the nanowire at the low simulated strain rates, and the symmetric property of the breaking position distribution becomes obvious with the strain rates increasing. However, the [111] nanowires in Figure 4d show the breaking position exhibits a single-peak distribution at the insensitive area of strain rate (I), and the symmetric distributions at the two ends of the nanowire gradually behave obvious with the strain rates increasing. From the influence of crystallographic anisotropy, we conclude the implied relationships in the scheme of Figure 1. When the symmetric stretching of the nanowire is applied at the low strain rates (less than 0.26% ps^-1^), micro-atomic fluctuation in equilibrium state brings the system enough ability to exhibit anisotropic characters of the crystal structures. Therefore, different deformation mechanisms for each crystallographic orientation exhibit various macro-breaking position distributions of the nanowires at low strain rates. When the symmetric stretching of the nanowire is applied at the high strain rates (larger than 3.54% ps^-1^), strong symmetric shocks dominate the deformation and breaking at the two ends of the nanowires. And the micro-atomic fluctuation has not enough ability to exhibit anisotropic characters of the crystal structures, thus, symmetric stretching results in macro-breaking position distributions at the two ends of nanowires.

For the microscopic behaviors of atoms under mechanical shocks, Holid and co-workers [49-51], Kadau et al. [52], and Bringa et al. [24] studied the shock wave propagation in solid materials and demonstrated its existence in nanoscale materials. Koh et al. [18,19] and Liu et al. [25] used the strain wave propagation theory to predict the breaking position of the nanowires. The theory could be stated that, if shock is involved, the longitudinal shock wave velocity can be derived from the simplified wave equation given by *U*s = (*Y*/*ρ*)^1/2^, *Y *is the Young's modulus and *ρ *is the average density of solid materials, which is estimated to be 8,900 kg/m^3^ for copper, and the most probable breaking position of the nanowires could be predicted and interpreted with the shock wave propagation theory, using the shock wave propagation distance *d *= *U*s × *t *= (*Y*/*ρ*)^1/2^ × *t*, and *t *is the required time to attain atomic break.

The microscopic mechanism of shock wave propagation in solids is inherently complex because the plastic flow is governed by the creation and motion of defects in the deformation of nanoscale materials. Meanwhile, with the statistical analysis of samples, we can find it is difficult to calculate exactly the fixed breaking position using the above shock wave propagation equation, especially for the breaking in the distributions of [100], [110], and [111] crystallographic orientations at the insensitive area of strain rates, and the uncertainty of [100] at the transitional area of strain rates. From microscopic viewpoint, mechanical shock is induced by symmetric stretching at different strain rates, and different mechanical shocks and anisotropies could affect the micro-atomic fluctuation, which could induce different ways of shock wave propagation. Mechanical shocks can disrupt the lattice order in the tensile deformation of the nanowires, attributing the concentrated and dispersed energy in shock wave propagation which is converted to the atomic kinetic energy, so the atomic bonds will break when the activated atoms have enough energy to overcome the atomic cohesive energy. At low strain rates, symmetric stretching retains the relative order lattice in the equilibrium state. In this state, micro-atomic fluctuation in anisotropic crystal structures mainly affect shock wave propagation in the stretching processes of the [100], [110], and [111] copper nanowires. Different styles of shock wave propagation for each crystallographic orientation make macro-breaking position distributions with various characters. At high strain rates, symmetric stretching in non-equilibrium state brings a large stress gradient, which induces the difficulty of shock wave propagation, so shock waves overlap at the two ends of the nanowires, which tend to break at the two ends without anisotropic behaviors. Thus, macro-breaking position distributions at the two ends of the nanowires show symmetric characters at high strain rates.

## Conclusion

In summary, we have simulated the [100], [110], and [111] single-crystal copper nanowires subjected to symmetric stretching at strain rates from 0.01 to 7.69% ps^-1^, and we have studied the deformation behaviors, mechanical properties, and their breaking position distributions. We find that: (i) at low strain rates, the [100], [110], and [111] crystallographic orientations behave as three deformation mechanisms, slippage, reconstruction, and rotation, respectively. Whereas, high strain rates easily induce their local melted structures at two ends of the nanowires; (ii) for the effect of strain rate on the mechanical properties of [100], [110], and [111] crystallographic orientations, [100] is obvious, [110] is not obvious, and [111] is between of them; (iii) the macro-breaking position distributions reflect the micro-atomic fluctuation during the symmetric stretching applied on the nanowires. When the strain rate is less than 0.26% ps^-1^, macro-breaking position distributions exhibit the structural anisotropy. However, the anisotropy is not obvious when the strain rate is larger than 3.54% ps^-1^ because of the strong symmetric shocks.

## Abbreviations

AFM: atomic force microscopy; EAM: embedded-atom method; FCC: face-centered cubic; MCBJ: mechanically controllable break junctions; MD: molecular dynamics; MPBP: most probable breaking position; NEMS: nanoelectromechanical system; STM: scanning tunneling microscopy; TEM: transmission electron microscope.

## Competing interests

The authors declare that they have no competing interests.

## Authors' contributions

FW carried out the simulations, participated in the design of the study, performed the statistical analysis and drafted the manuscript. YG and TZ participated in discussions. JZ conceived of the study, and participated in its design and coordination. All authors read and approved the final manuscript.

## Supplementary Material

Additional file 1 **Video S1**. A movie of deformation behavior of the [100] single-crystal copper nanowire at the strain rate of 0.01% ps^-1^. Click here for file

Additional file 2 **Video S2**. A movie of deformation behavior of the [100] single-crystal copper nanowire at the strain rate of 1.54% ps^-1^. Click here for file

Additional file 3 **Video S3**. A movie of deformation behavior of the [100] single-crystal copper nanowire at the strain rate of 6.16% ps^-1^. Click here for file

Additional file 4 **Video S4**. A movie of deformation behavior of the [110] single-crystal copper nanowire at the strain rate of 0.01% ps^-1^. Click here for file

Additional file 5 **Video S5**. A movie of deformation behavior of the [110] single-crystal copper nanowire at the strain rate of 1.54% ps^-1^. Click here for file

Additional file 6 **Video S6**. A movie of deformation behavior of the [110] single-crystal copper nanowire at the strain rate of 6.16% ps^-1^. Click here for file

Additional file 7 **Video S7**. A movie of deformation behavior of the [111] single-crystal copper nanowire at the strain rate of 0.01% ps^-1^. Click here for file

Additional file 8 **Video S8**. A movie of deformation behavior of the [111] single-crystal copper nanowire at the strain rate of 1.54% ps^-1^. Click here for file

Additional file 9 **Video S9**. A movie of deformation behavior of the [111] single-crystal copper nanowire at the strain rate of 6.16% ps^-1^. Click here for file

Additional file 10 **Figure S1**. The maximum average potential energy per atom plotted against strain rates for the [100], [110], and [111] single-crystal copper nanowires. Figure S2 The representative stress-strain relationship of the [110] copper nanowire at the strain rates of 0.01, 1.54, and 6.16% ps^-1^. Figure S3 The representative stress-strain relationship of the [111] copper nanowire at the strain rates of 0.01, 1.54, and 6.16% ps^-1^. **Stress-strain response for [110] and [111] crystallographic orientation** Figure S2 in Additional file 1 shows the typical stress-strain responses of the single-crystal copper nanowire along the [110] orientation from the initial equilibrium state to complete breakage at the strain rates of 0.01, 1.54, and 6.16% ps^-1^. The stress-strain responses in Figure S2 (Additional file 1) correspond to the representative deformation behaviors of the [110] copper nanowire in Videos S4, S5, and S6 of Additional files 5, 6, and 7, respectively (see Videos S4-S6 in Additional files 5, 6, 7). For all the stress-strain responses, stress increases linearly with an increase in strain before the first yield point. After the first yield point, the stress decreases abruptly indicating the nanowire undergoes the plastic deformation and the irreversible deformation begins. Subsequently, the yield cycles repeat continuously until the final breaking of the nanowire. From Figure S2 in Additional file 1, we can find that the first yield strain increases from 0.057 to 0.075 when the strain rates increase from 0.01 to 6.16% ps^-1^, moreover, the breaking strain also increases from 0.433 to 1.286. In general, the stress-strain curve could reflect the tensile process and deformation mechanism, which depend on crystallographic orientation and strain rate. As shown in Video S4 of Additional file 5, the [110] nanowire prefers to maintain the crystallographic structure at low strain rate of 0.01% ps^-1^. The neck appears abruptly with the strain increasing, and then the nanowire breaks accompanying with a few atoms in the disorder movement. It is because the preferred slip directions are identical to the tensile direction so that the system has no ability to get the atomic rearrangement at lower strain rates. Thus, the stress-strain curve behaves sharp stress peaks and few yield cycles in Figure S2a (Additional file 1). At the low strain rate, the copper nanowire remains a better crystal structures due to weak mechanical shocks. Rising strain rate would increase the atomic thermal motion, which facilitates the ductility of the materials during stretching. Therefore, the stress-strain response embodies the dependence of mechanical properties on the strain rates. For the stress-strain response at the middle strain rate of 1.54% ps^-1^, the stress peaks of the yield cycles in Figure S2b (Additional file 1) are not as sharp as the ones at low strain rate in Figure S2a (Additional file 1), and the tensile strain increases obviously at this condition. Meanwhile, Video S5 in Additional file 6 shows that a local lattice reconstruction becomes predominant after the first yield point, and necking takes place at these reconstruction positions. Unlike the low and middle strain rates, the stress-strain curve of the [110] orientation are separated by a large stress well at the strain rate of 6.16% ps^-1 ^(Figure S2c in Additional file 1). Before the first yielding point, the nanowire undergoes elastic stretching. After the first yielding point, a stress hardening over a relatively wide range of strain can be observed. Between the first and the second yielding points, a local lattice reconstruction process occurs and it spreads to the whole nanowire. In Figure S2c (Additional file 1), the stress-strain response corresponds to Video S6 in Additional file 7 which exhibits local disordered deformation and the superplasticity behaviors. With the tension strain increasing, it is more likely to break near the two ends of the nanowire. In comparison with the single-crystal copper nanowire along the [110] orientation, Figure S3 in Additional file 1 shows the typical stress-strain properties of copper nanowires stretched along the [111] orientation, which is simulated at the strain rates of 0.01, 1.54, and 6.16% ps^-1^. At the first stretching stage, the stress increases almost linearly for all the nanowires, and then reaches a critical point. In this process, the nanowires experience elastic deformation just like that of the [110] orientation. We can also find that both the yielding strain and the breaking strain increase with the strain rate increasing. However, [111] are lower than [110] at low and middle strain rates, whereas at high strain rate, [111] is higher than [110]. Click here for file
