# Supplementary material for: Shock-induced breaking of the nanowire with the dependence of crystallographic orientation and strain rate
Source: Nanoscale Res Lett. 2011 Apr 5;6(1):291. doi: 10.1186/1556-276X-6-291 (PMC3211357; doi:10.1186/1556-276X-6-291)
Supplement: Additional file 1 — Video S1. A movie of deformation behavior of the [100] single-crystal copper nanowire at the strain rate of 0.01% ps-1. [file 1556-276X-6-291-S1.DOC]

Supporting information

**Shock-induced breaking of the nanowire with the dependence of crystallographic orientation and strain rate**

Fenying Wang, Yajun Gao, Tiemin Zhu, and Jianwei Zhao*

Email: [zhaojw@nju.edu.cn](mailto:zhaojw@nju.edu.cn), Tel/Fax: +86-25-83596523

*Key Laboratory of Analytical Chemistry for Life Science, Ministry of Education, School of Chemistry and Chemical Engineering, Nanjing University, Nanjing 210008, P. R. China*

**Supplementary Figure Captions**

**Fig. S1** The maximum average potential energy per atom plotted against strain rates for the [100], [110] and [111] single-crystal copper nanowires

**Fig. S2** The representative stress-strain relationship of the [110] copper nanowire at the strain rates of 0.01%, 1.54% and 6.16% ps^-1^.

**Fig. S3** The representative stress-strain relationship of the [111] copper nanowire at the strain rates of 0.01%, 1.54% and 6.16% ps^-1^.

**Supplementary Multimedia Captions**

**Video S1** A movie of deformation behavior of the [100] single-crystal copper nanowire at the strain rate of 0.01% ps^-1^

**Video S2** A movie of deformation behavior of the [100] single-crystal copper nanowire at the strain rate of 1.54% ps^-1^

**Video S3** A movie of deformation behavior of the [100] single-crystal copper nanowire at the strain rate of 6.16% ps^-1^

**Video S4** A movie of deformation behavior of the [110] single-crystal copper nanowire at the strain rate of 0.01% ps^-1^

**Video S5** A movie of deformation behavior of the [110] single-crystal copper nanowire at the strain rate of 1.54% ps^-1^

**Video S6** A movie of deformation behavior of the [110] single-crystal copper nanowire at the strain rate of 6.16% ps^-1^

**Video S7** A movie of deformation behavior of the [111] single-crystal copper nanowire at the strain rate of 0.01% ps^-1^

**Video S8** A movie of deformation behavior of the [111] single-crystal copper nanowire at the strain rate of 1.54% ps^-1^

**Video S9** A movie of deformation behavior of the [111] single-crystal copper nanowire at the strain rate of 6.16% ps^-1^

**Fig. S1**

**
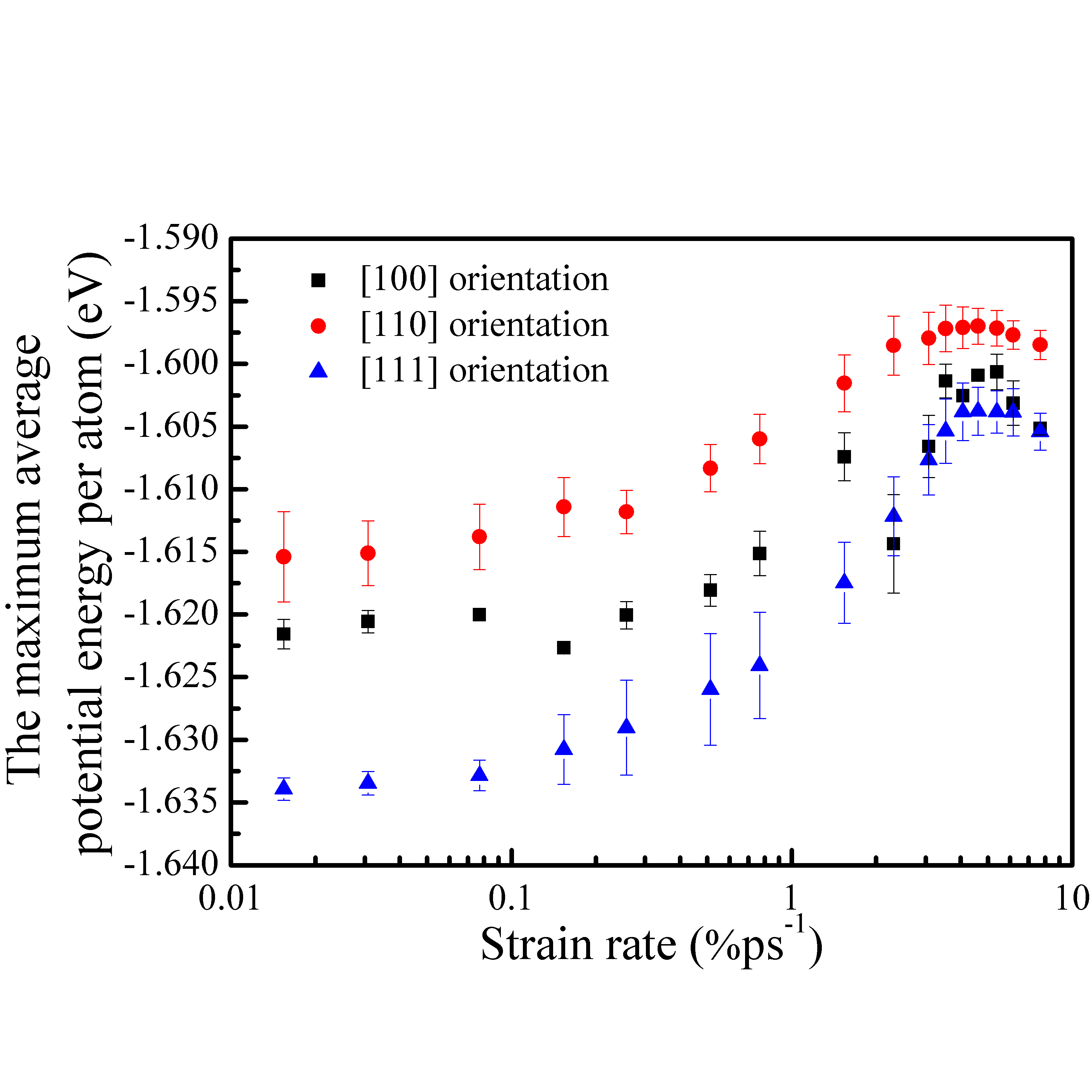
**

**Fig. S2**

**
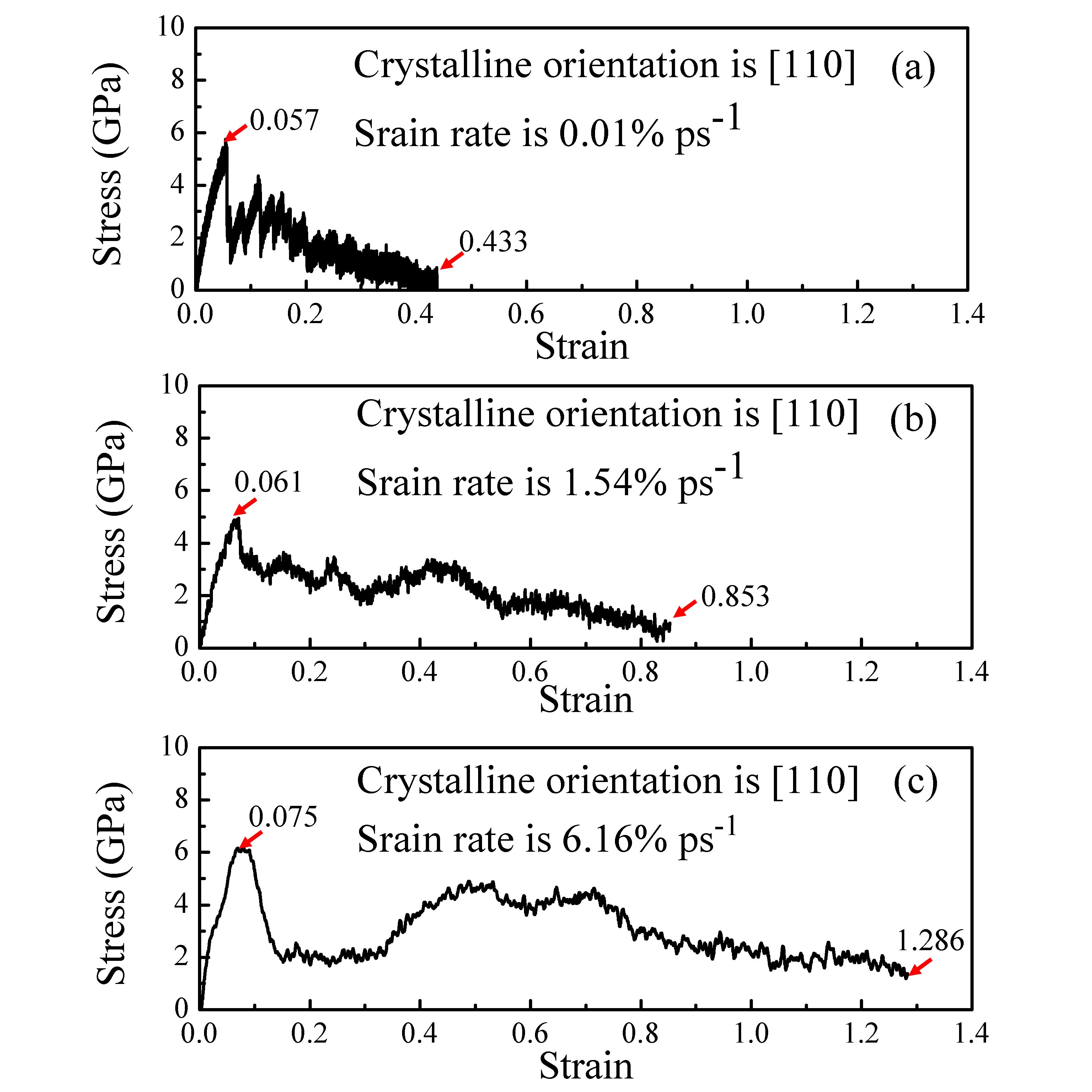
**

**Fig. S3**

**
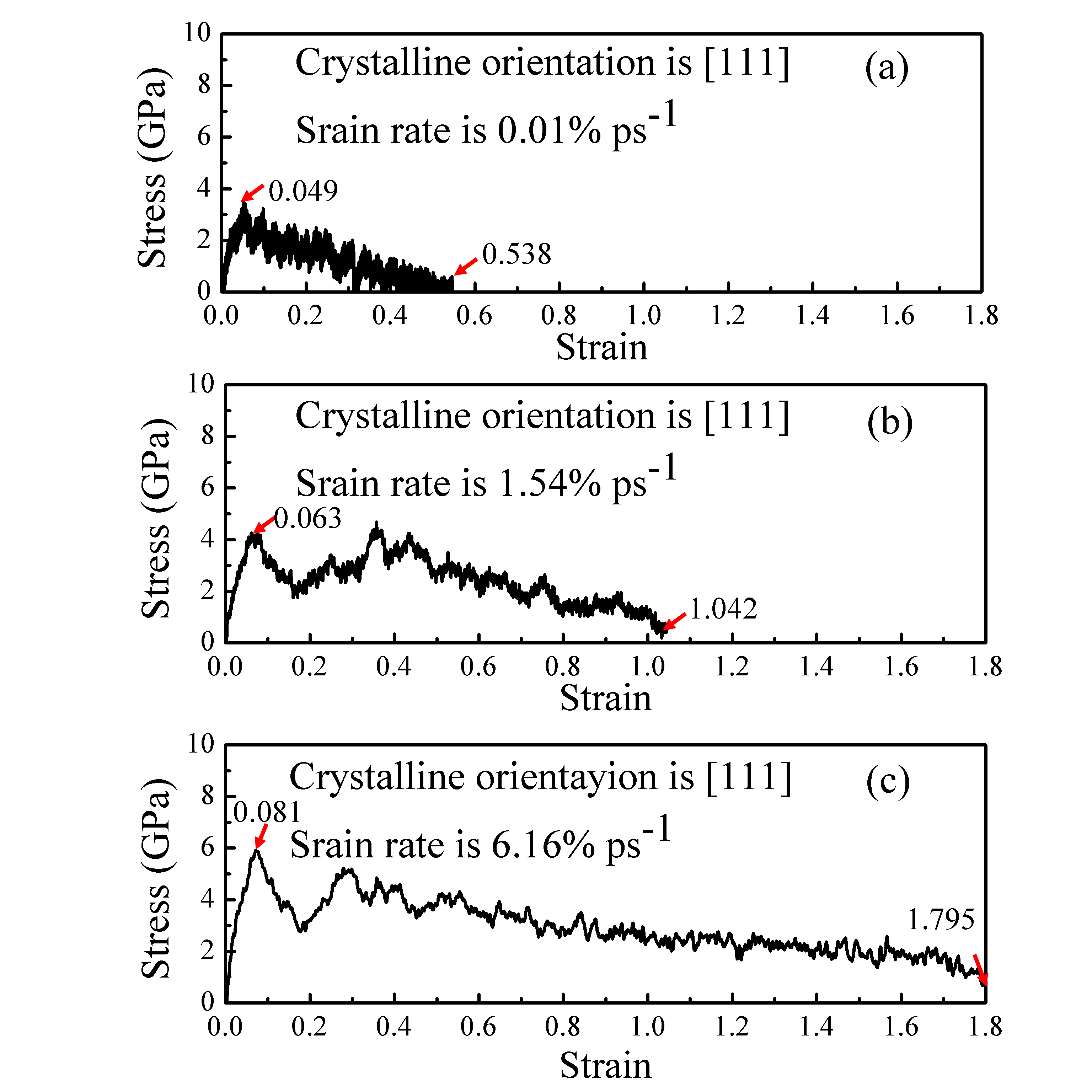
**

**Stress-strain response for [110] and [111] crystallographic orientation**

Fig. S2 give the typical stress-strain responses of the single-crystal copper nanowire along the [110] orientation from the initial equilibrium state to complete breakage at the strain rates of 0.01%, 1.54% and 6.16% ps^-1^. The stress-strain responses in Figure S2 correspond to the representative deformation behaviors of the [110] copper nanowire in Video S1-S3, respectively (see Supplementary Video S4-S6). For all the stress-strain responses, stress increases linearly with an increase in strain before the first yield point. After the first yield point, the stress decreases abruptly indicating the nanowire undergoes the plastic deformation and the irreversible deformation begins. Subsequently, the yield cycles repeat continuously until the final breaking of the nanowire.

From Fig. S2, we can find that the first yield strain increases from 0.057 to 0.075 when the strain rates increase from 0.01% to 6.16% ps^-1^, moreover, the breaking strain also increases from 0.433 to 1.286. In general, the stress-strain curve could reflect the tensile process and deformation mechanism, which depend on crystallographic orientation and strain rate. As shown in Video S4, the [110] nanowire prefers to maintain the crystallographic structure at low strain rate of 0.01% ps^-1^. The neck appears abruptly with the strain increasing, and then the nanowire breaks accompanying with a few atoms in the disorder movement. It is because the preferred slip directions are identical to the tensile direction so that the system has no ability to get the atomic rearrangement at lower strain rates. Thus, the stress-strain curve behaves sharp stress peaks and few yield cycles in Fig. S2(a).

At the low strain rate, the copper nanowire remains a better crystal structures due to weak mechanical shocks. Rising strain rate would increase the atomic thermal motion, which facilitates the ductility of the materials during stretching. Therefore, the stress-strain response embodies the dependence of mechanical properties on the strain rates. For the stress-strain response at the middle strain rate of 1.54% ps^-1^, the stress peaks of the yield cycles in Fig. S2(b) are not as sharp as the ones at low strain rate in Fig. S2(a), and the tensile strain increases obviously at this condition. Meanwhile, Video S5 shows that a local lattice reconstruction becomes predominant after the first yield point, and necking takes place at these reconstruction positions. Unlike the low and middle strain rates, the stress-strain curve of the [110] orientation are separated by a large stress well at the strain rate of 6.16% ps^-1^ (Fig. S2(c)). Before the first yielding point, the nanowire undergoes elastic stretching. After the first yielding point, a stress hardening over a relatively wide range of strain can be observed. Between the first and second yielding points, a local lattice reconstruction process occurs and it spreads to the whole nanowire. In Fig. S2(c), the stress-strain response corresponds to Video S6 which exhibits local disordered deformation and the superplasticity behaviors. With the tension strain increasing, it is more likely to break near the two ends of the nanowire.

In comparison with the single-crystal copper nanowire along the [110] orientation, Figure S3 shows the typical stress-strain properties of copper nanowires stretched along the [111] orientation, which is simulated at the strain rates of 0.01%, 1.54% and 6.16% ps^-1^. At the first stretching stage, the stress increases almost linearly for all the nanowires, and then reaches a critical point. In this process, the nanowires experience elastic deformation just like that of the [110] orientation. We can also find that both the yielding strain and the breaking strain increase with the strain rate increasing. However, [111] are lower than [110] at low and middle strain rates, whereas, at high strain rate, [111] is higher than [110].
