# Supplementary figures and images for: Shock-induced breaking of the nanowire with the dependence of crystallographic orientation and strain rate
Source: Nanoscale Res Lett. 2011 Apr 5;6(1):291. doi: 10.1186/1556-276X-6-291 (PMC3211357; doi:10.1186/1556-276X-6-291)

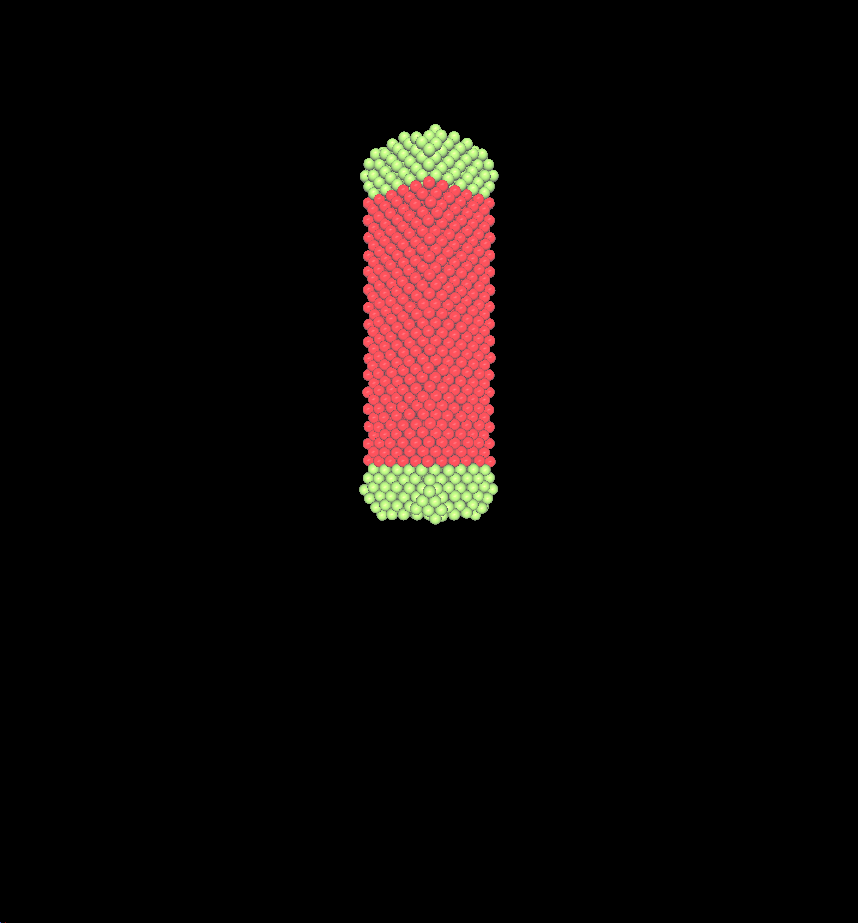

Supplement: Additional file 2 — Video S2. A movie of deformation behavior of the [100] single-crystal copper nanowire at the strain rate of 1.54% ps-1. [file 1556-276X-6-291-S2.GIF]

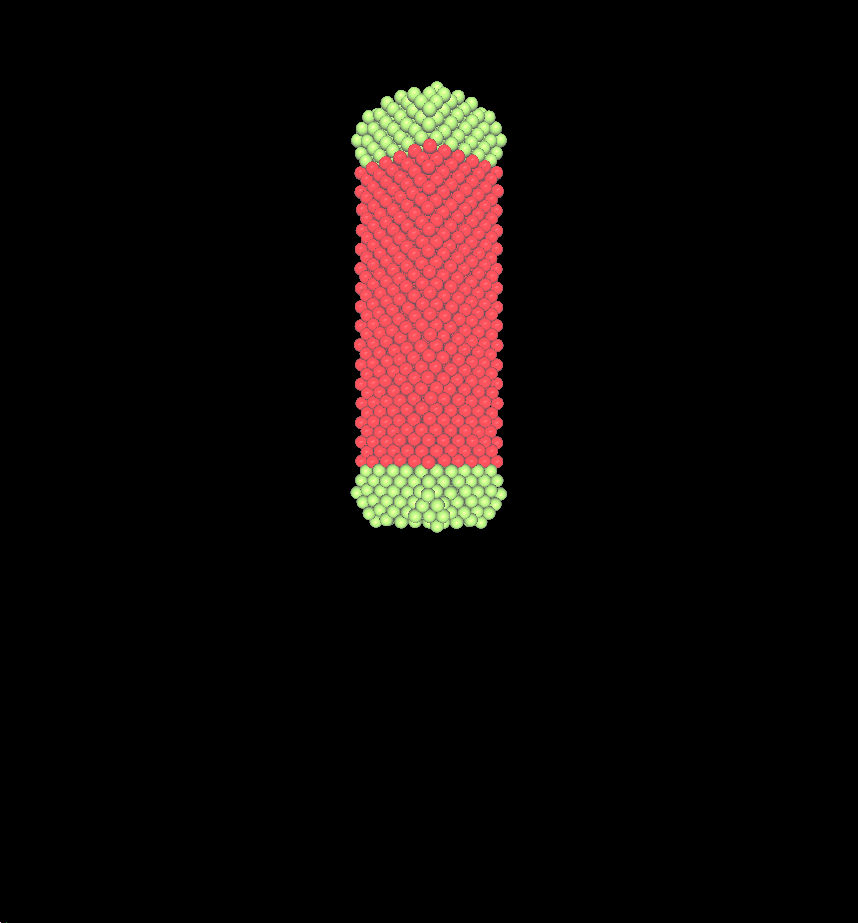

Supplement: Additional file 3 — Video S3. A movie of deformation behavior of the [100] single-crystal copper nanowire at the strain rate of 6.16% ps-1. [file 1556-276X-6-291-S3.GIF]

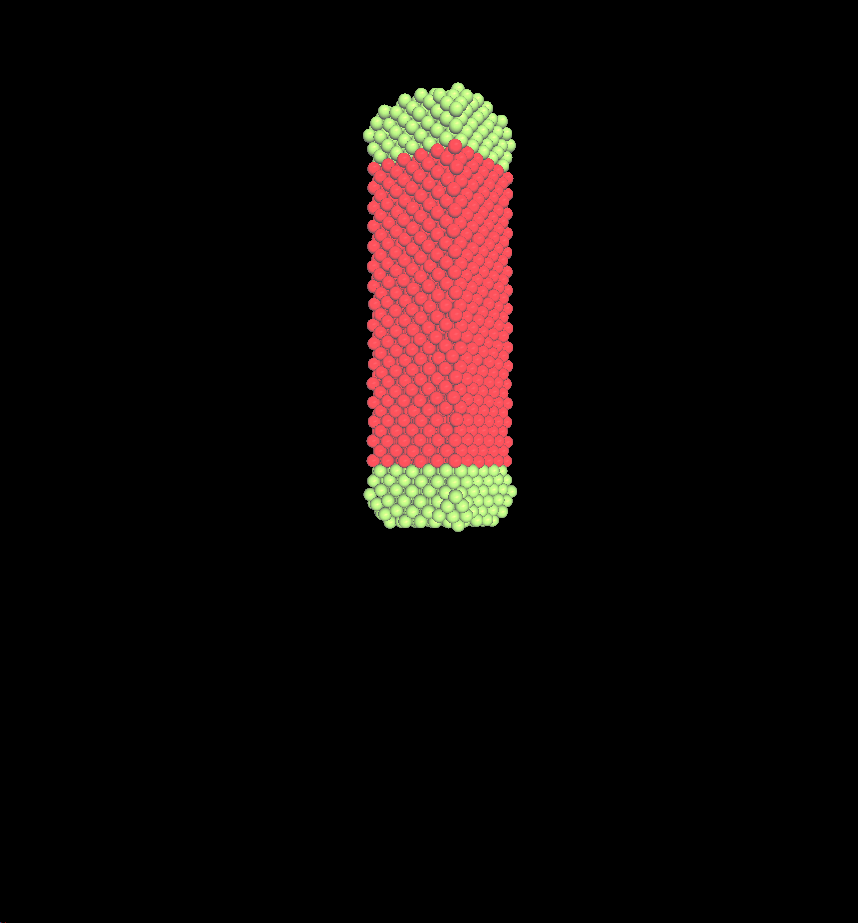

Supplement: Additional file 4 — Video S4. A movie of deformation behavior of the [110] single-crystal copper nanowire at the strain rate of 0.01% ps-1. [file 1556-276X-6-291-S4.GIF]

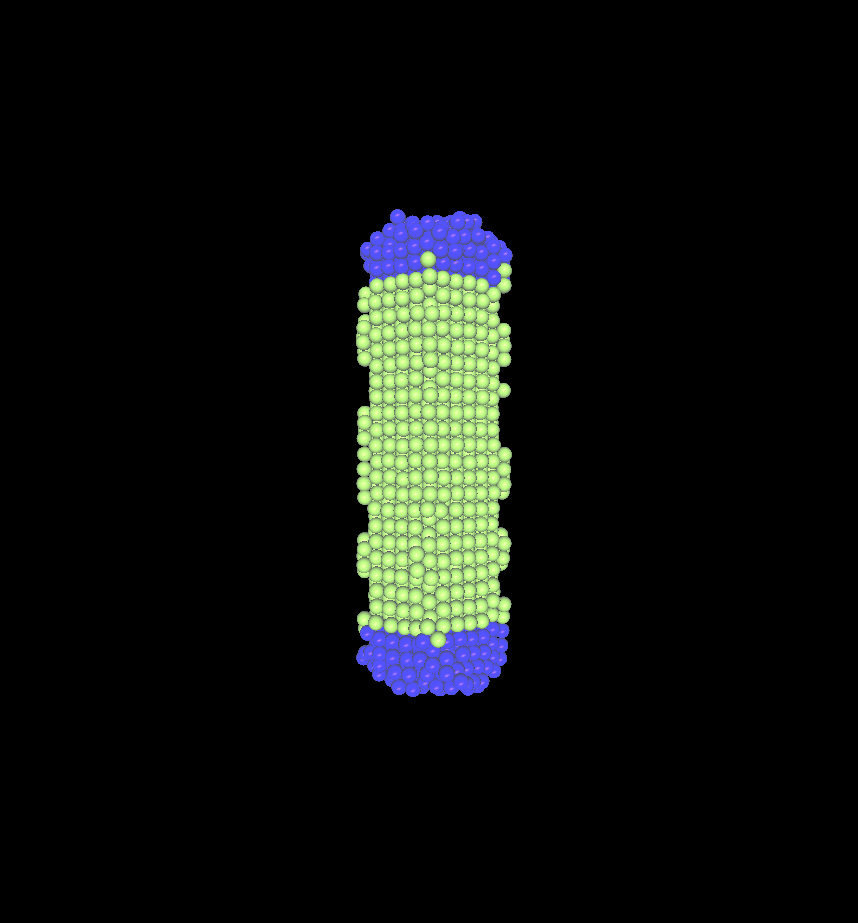

Supplement: Additional file 5 — Video S5. A movie of deformation behavior of the [110] single-crystal copper nanowire at the strain rate of 1.54% ps-1. [file 1556-276X-6-291-S5.GIF]

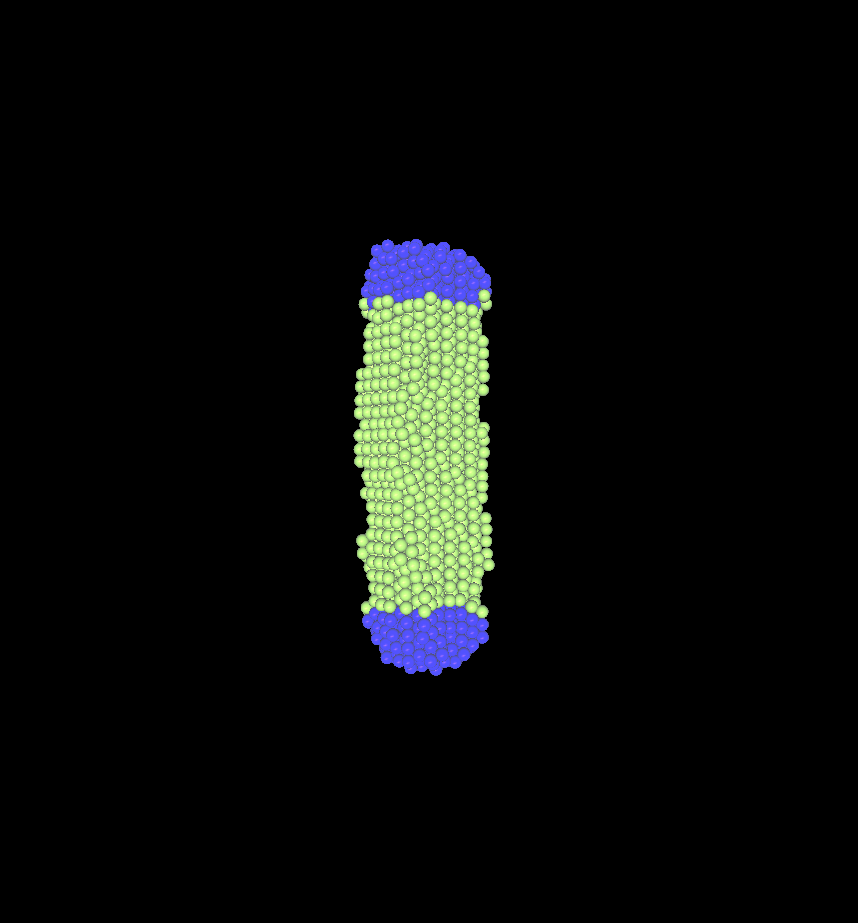

Supplement: Additional file 6 — Video S6. A movie of deformation behavior of the [110] single-crystal copper nanowire at the strain rate of 6.16% ps-1. [file 1556-276X-6-291-S6.GIF]

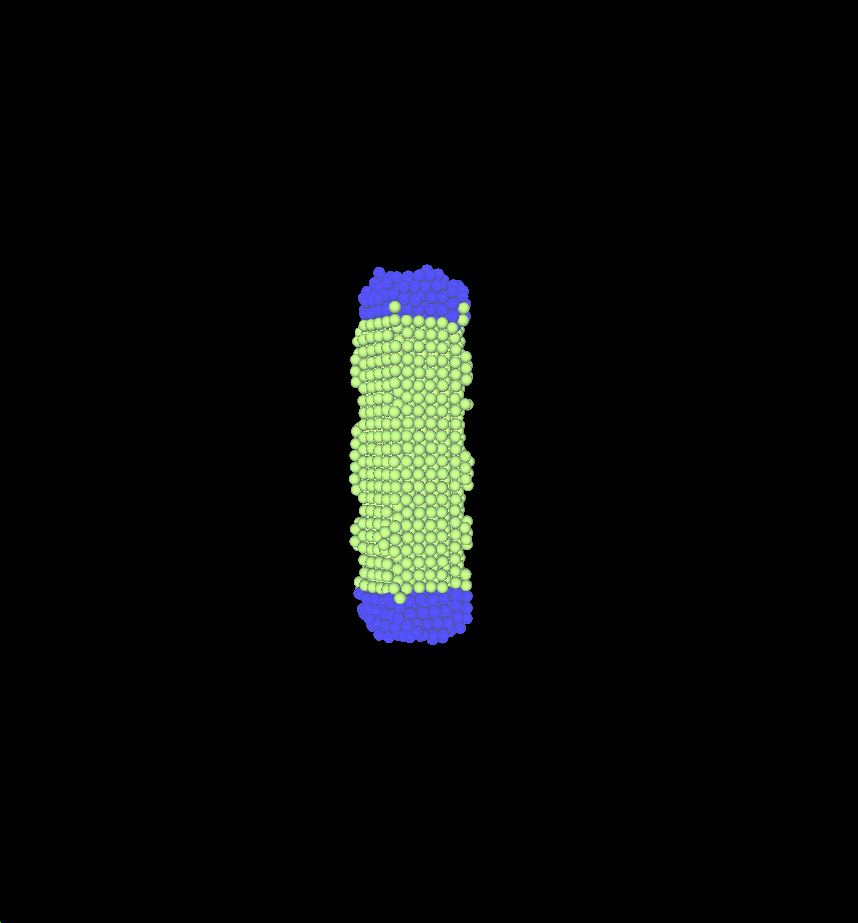

Supplement: Additional file 7 — Video S7. A movie of deformation behavior of the [111] single-crystal copper nanowire at the strain rate of 0.01% ps-1. [file 1556-276X-6-291-S7.GIF]

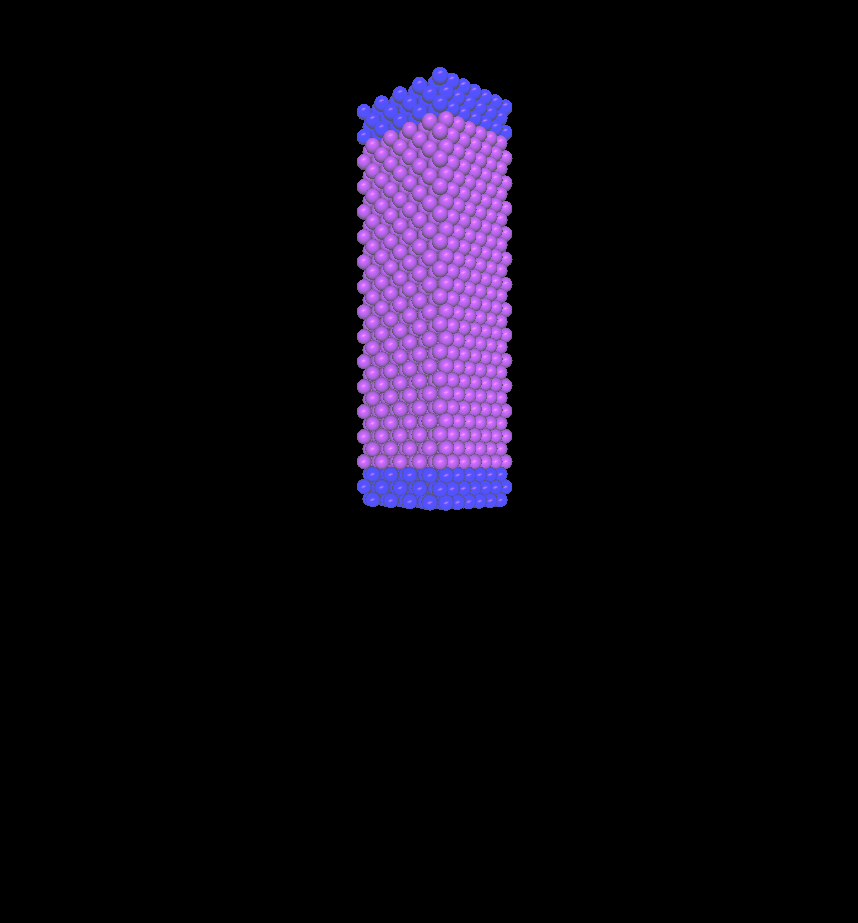

Supplement: Additional file 8 — Video S8. A movie of deformation behavior of the [111] single-crystal copper nanowire at the strain rate of 1.54% ps-1. [file 1556-276X-6-291-S8.GIF]

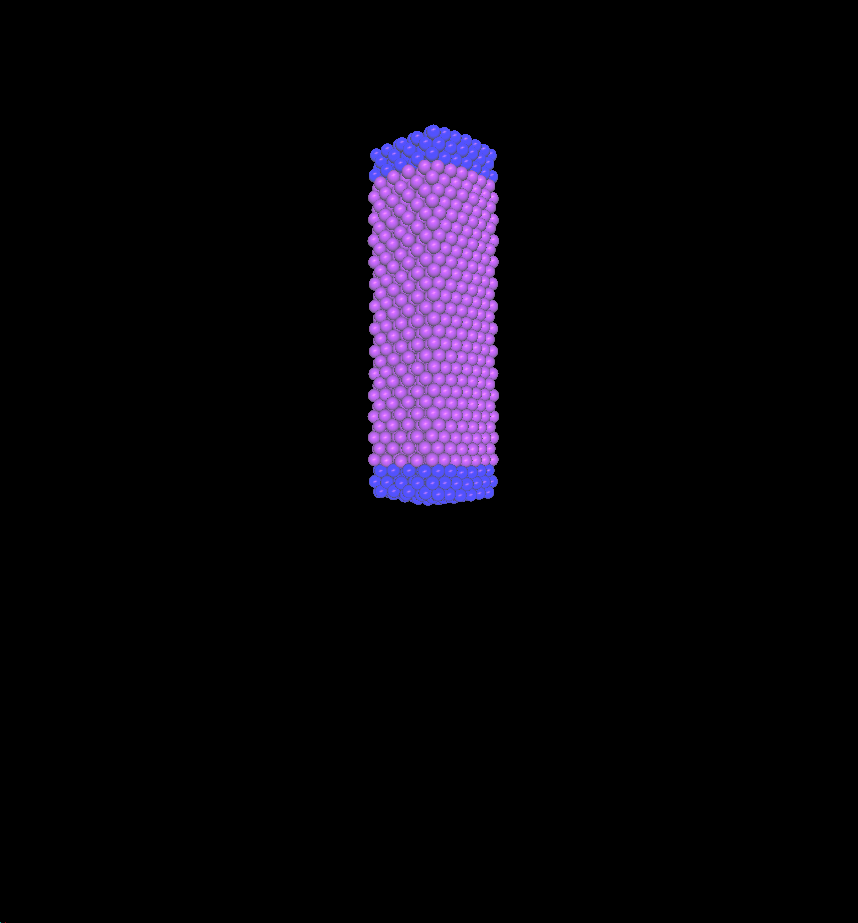

Supplement: Additional file 9 — Video S9. A movie of deformation behavior of the [111] single-crystal copper nanowire at the strain rate of 6.16% ps-1. [file 1556-276X-6-291-S9.GIF]

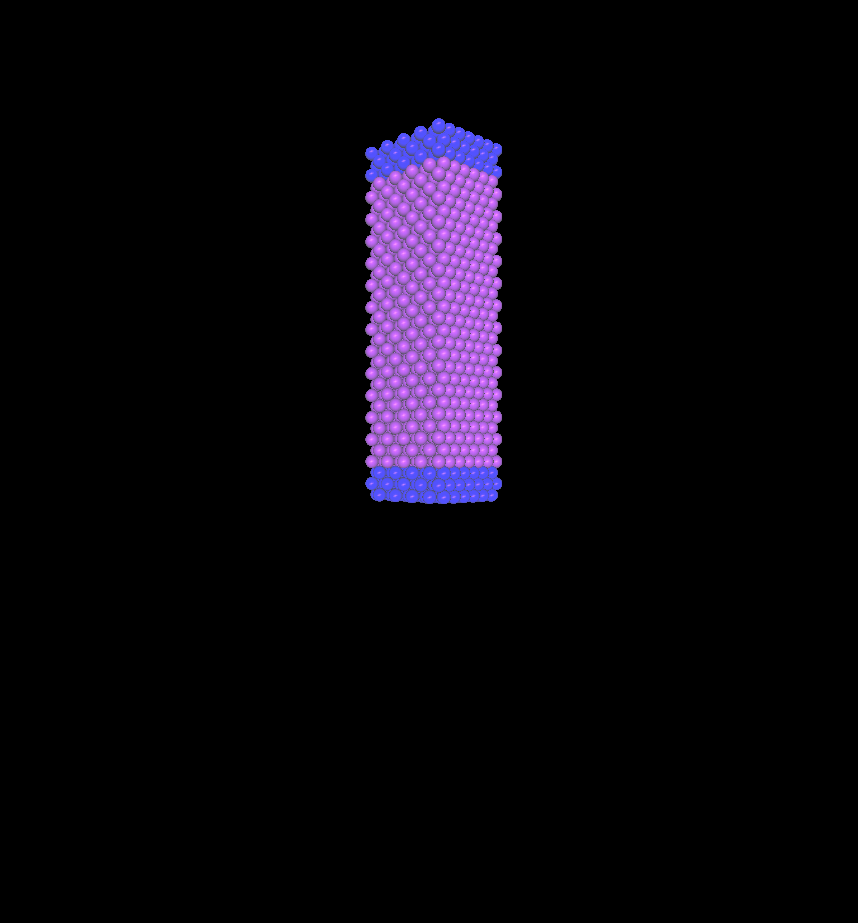

Supplement: Additional file 10 — Figure S1. The maximum average potential energy per atom plotted against strain rates for the [100], [110], and [111] single-crystal copper nanowires. Figure S2 The representative stress-strain relationship of the [110] copper nanowire at the strain rates of 0.01, 1.54, and 6.16% ps-1. Figure S3 The representative stress-strain relationship of the [111] copper nanowire at the strain rates of 0.01, 1.54, and 6.16% ps-1. Stress-strain response for [110] and [111] crystallographic orientation Figure S2 in Additional file 1 shows the typical stress-strain responses of the single-crystal copper nanowire along the [110] orientation from the initial equilibrium state to complete breakage at the strain rates of 0.01, 1.54, and 6.16% ps-1. The stress-strain responses in Figure S2 (Additional file 1) correspond to the representative deformation behaviors of the [110] copper nanowire in Videos S4, S5, and S6 of Additional files 5, 6, and 7, respectively (see Videos S4-S6 in Additional files 5, 6, 7). For all the stress-strain responses, stress increases linearly with an increase in strain before the first yield point. After the first yield point, the stress decreases abruptly indicating the nanowire undergoes the plastic deformation and the irreversible deformation begins. Subsequently, the yield cycles repeat continuously until the final breaking of the nanowire. From Figure S2 in Additional file 1, we can find that the first yield strain increases from 0.057 to 0.075 when the strain rates increase from 0.01 to 6.16% ps-1, moreover, the breaking strain also increases from 0.433 to 1.286. In general, the stress-strain curve could reflect the tensile process and deformation mechanism, which depend on crystallographic orientation and strain rate. As shown in Video S4 of Additional file 5, the [110] nanowire prefers to maintain the crystallographic structure at low strain rate of 0.01% ps-1. The neck appears abruptly with the strain increasing, and then the nanowire breaks accomp [file 1556-276X-6-291-S10.GIF]
